# Small Finger Snapping due to Retinacular Ligament Injury at the Level of Proximal Interphalangeal Joint: A Case Report

**DOI:** 10.1097/MD.0000000000000996

**Published:** 2015-06-19

**Authors:** Young-Keun Lee, Jun-Mo Lee, Malrey Lee

**Affiliations:** From the Department of Orthopedic Surgery, Chonbuk National University Hospital (Y-KL, J-ML); and The Research Center for Advanced Image and Information Technology, School of Electronics and Information Engineering, ChonBuk National University, JeonJu, ChonBuk, Republic of Korea (ML).

## Abstract

Pathological snapping secondary to posttraumatic subluxation of the extensor tendon at proximal interphalangeal joint (PIPJ) of the finger is rare. Here, we want to describe a patient with snapping of the left small finger at PIPJ due to retinacular ligament injury.

A 24-year-old man was admitted because of a 5-year history of a snapping sound in the left small finger. On examination, the radial side lateral band of the small finger was dislocated volarly at the level PIPJ with flexion of >50°, which was clearly felt over the skin. There was an obvious snapping sound at the time of dislocation. There was no specific radiographic abnormality. With the patient under regional anesthesia, exploration through a zigzag skin incision over the dorsum of the PIPJ revealed that the retinacular ligament complex was injured. We also found a partial tear in PIPJ capsule, through the incision of the injured retinacular ligament complex. We repaired the joint capsule and retinacular ligament complex with prolene 4–0. Postoperatively the small finger was immobilized in a below-elbow plaster splint with full extension of the fingers for 1 week, then dynamic splinting was advised for another 5 weeks and unrestricted full active motion was allowed at the 6th week.

At the 6-month follow-up, the patient had regained full range of motion with no discomfort, and there was no sign of recurrence.

We stress that when there is snapping over the dorsum of the PIPJ of the finger, the clinician should suspect rupture of the retinacular ligaments, especially in minor trauma patients. Primary repair of retinacular ligaments and dynamic splinting provided satisfactory results without recurrence in our patient.

## INTRODUCTION

Snapping fingers at the metacarpophalangeal joint has been reported frequently.^1,2^ However, pathological snapping secondary to posttraumatic subluxation of the extensor tendon at proximal interphalangeal joint (PIPJ) of the finger is rare. To our knowledge, there is just 1 report in the English-language literature.^3^ Here, we describe a patient with snapping of the left small finger at PIPJ due to retinacular ligament injury.

## CONSENT

The patient signed informed consent for the publication of this case report and any accompanying image. The ethical approval of this study was waived by the ethics committee of Chonbuk National University Hospital, because this study was case report and the number of patients was less than 3.

## CASE REPORT

A 24-year-old man was admitted because of a 5-year history of a snapping sound in the left small finger, since he had suffered sprain trauma in his small finger while playing basketball. He had a history of an operation for A1 pulley release in the same finger under the diagnosis of trigger finger due to a snapping sound 4 years earlier at another hospital. However, the snapping sound did not resolve after that operation.

On examination, the radial side lateral band of the small finger was dislocated volarly at the level PIPJ with flexion of >50°, which was clearly felt over the skin. There was an obvious snapping sound at the time of dislocation. There was no specific radiographic abnormality.

**FIGURE 1:**
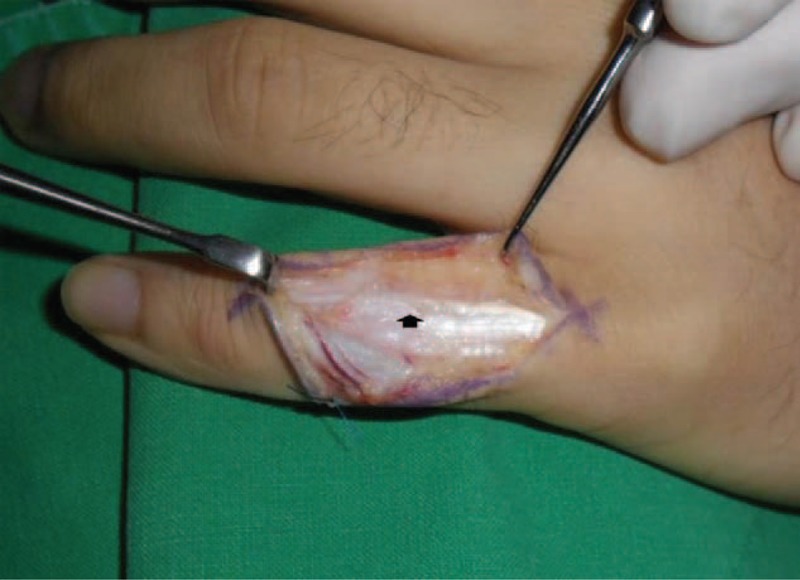
Surgical exploration revealed a partially injured and attenuated retinacular ligament complex from the distal part of the lamina intertendineum between the central slip and the lateral band to the triangular retinacular ligament at the radial side (arrow).

**FIGURE 2:**
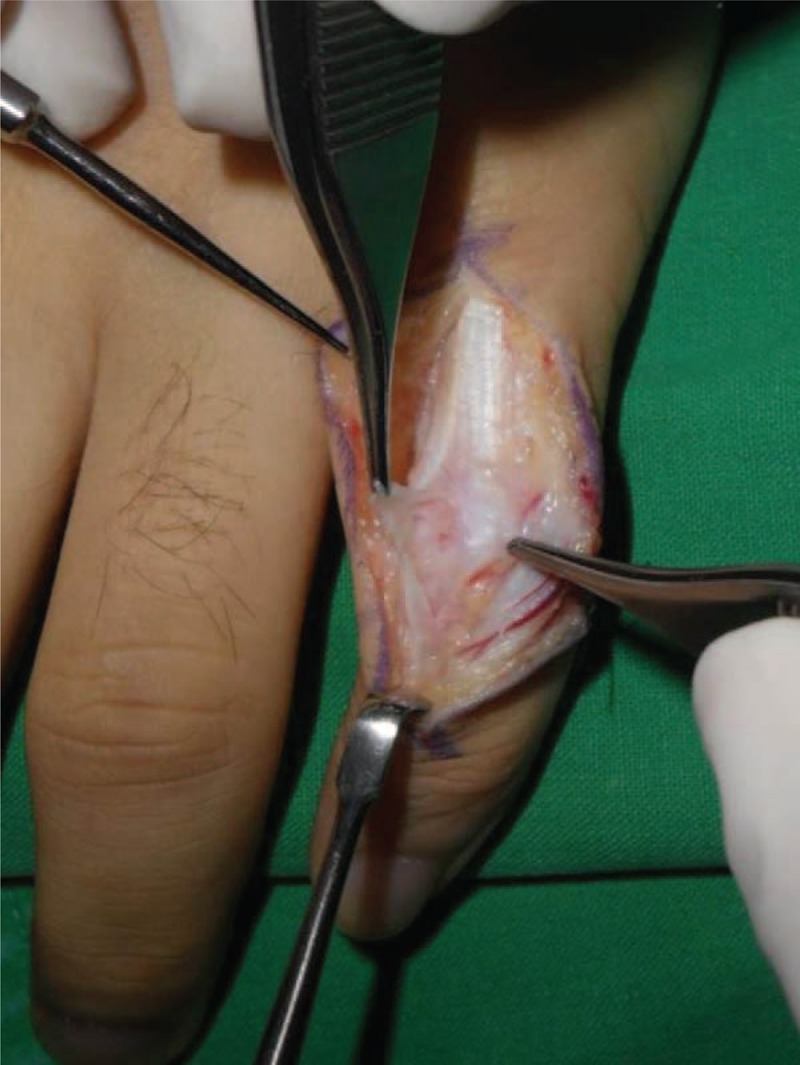
Partially damaged PIPJ capsule seen through an incision in the attenuated retinacular ligament complex. PIPJ = proximal interphalangeal joint.

**FIGURE 3:**
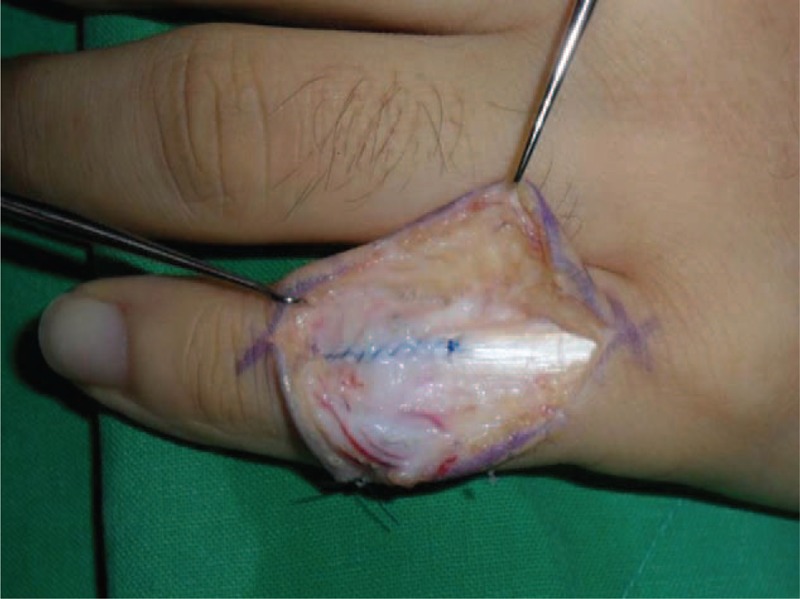
Damaged PIPJ capsule and retinacular ligaments were sutured with 4/0 prolene. PIPJ = proximal interphalangeal joint.

**FIGURE 4:**
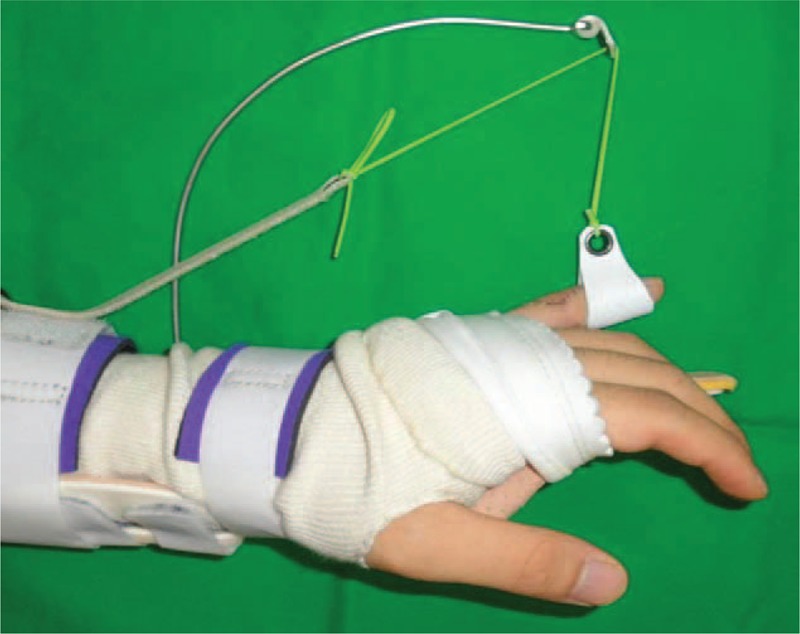
Postoperative dynamic splinting appearance.

With the patient under regional anesthesia, exploration through a zigzag skin incision over the dorsum of the PIPJ revealed that the retinacular ligament complex was injured and was attenuated from the distal part of the lamina intertendineum between the central slip and the lateral band to the triangular ligament at the radial side (Figure 1). We also found a partial tear in PIPJ capsule, through the incision of the injured retinacular ligament complex (Figure 2). We repaired the joint capsule by means of a simple interrupted suture with prolen 4-0 and carefully retinacular ligament complex by means of a running suture along the ruptured margin without debridement with prolene 4-0 (Figure 3). Postoperatively the small finger was immobilized in a below-elbow plaster splint with full extension of the fingers for 1 week, then dynamic splinting was advised for another 5 weeks and unrestricted full active motion was allowed at the 6th week (Figure 4).

**FIGURE 5:**
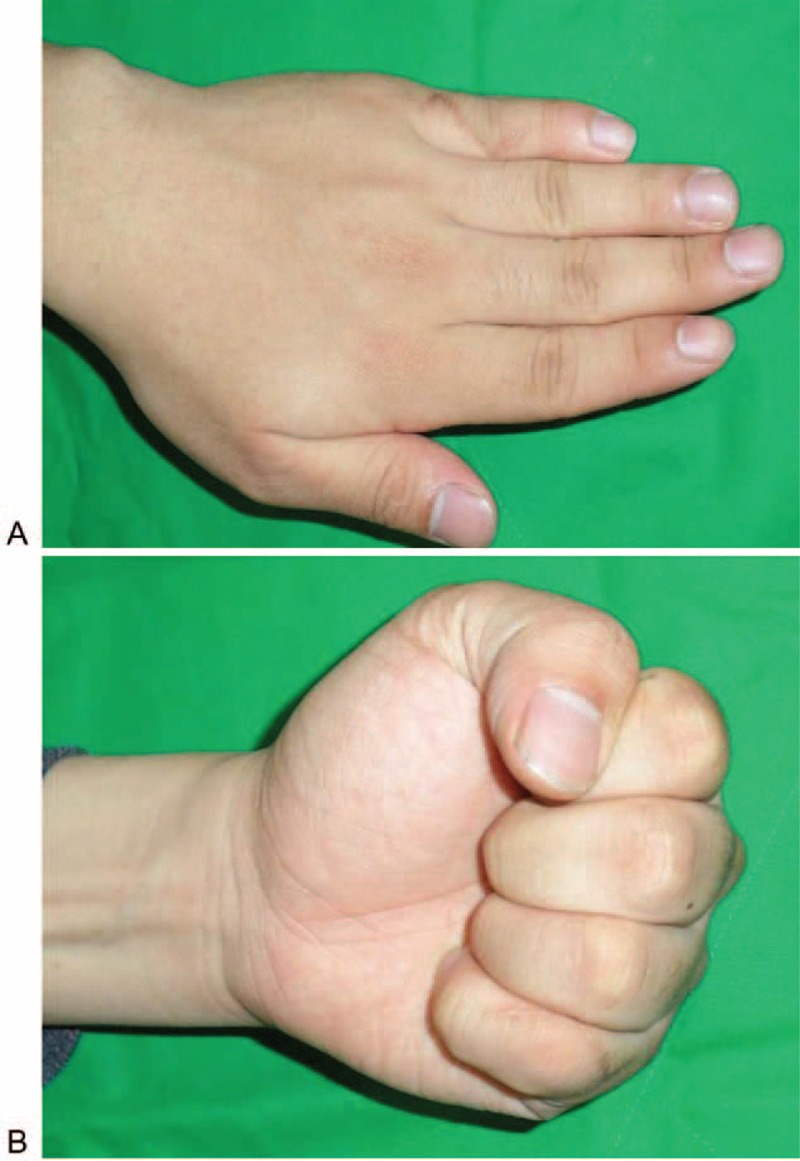
(A, B) At Follow-up, 6 months later, he had regained a normal range of motion of the small finger without snapping.

At the 6-month follow-up, the patient had regained full range of motion with no discomfort, and there was no sign of recurrence (Figure 5).

## DISCUSSION

In terms of the PIPJ extensor mechanism, dorsal and volar migration of the lateral band has a relatively simple mechanical basis; however, it should be controlled by triangular ligament and the transverse retinacular ligament.^4,5^ The triangular ligament provides stability to the lateral bands preventing palmar subluxation during PIPJ flexion. The transverse retinacular ligament prevents dorsal subluxation of the lateral band. Thus, the elastic balance of the triangular ligament and transverse retinacular ligament is important for normal functioning of the lateral bands. As in this case, injury of this elastic tissue as a result of trauma can be cause of a finger deformity, such as a boutonniere deformity and swan neck deformity, which may include snapping of the lateral band. There are just 2 references in English about the finger snapping at the PIPJ. One was caused by solitary periosteal chondroma,^6^ and the other by slipping of the lateral band.^3^ Ikeda et al^3^ explained the causes of snapping in their case from 2 points of view; 1 was an anatomical cause and the other was a traumatic cause, rupturing the transverse retinacular ligament due to minor trauma as a direct cause of finger snapping. In the case of our patient, the anatomy of the PIPJ was apparently normal, so we concluded that the injury of the retinacular ligament complex around the PIPJ was a direct cause of the snapping. The surgical treatment for this type of injury remains controversial. Ikeda et al^3^ cut the lateral band and sutured to the central slip for treatment. We think that this method caused a deformation of the relationship of the normal extensor tendons. We wanted to achieve recovery of the elasticity of retinacular ligaments without deformation of normal anatomy, so we repaired the attenuated retinacular ligaments. The procedure we performed was simple and effective.

## CONCLUSION

We stress that when there is snapping over the dorsum of the PIPJ of the finger, the clinician should suspect rupture of the retinacular ligaments, especially in minor trauma patients. Knowledge of the anatomical and functional characteristics of these structures is important when treating these conditions. Primary repair of retinacular ligaments and dynamic splinting provided satisfactory results without recurrence in our patient.
